# Identification of self-interacting proteins by exploring evolutionary information embedded in PSI-BLAST-constructed position specific scoring matrix

**DOI:** 10.18632/oncotarget.12517

**Published:** 2016-10-08

**Authors:** Ji-Yong An, Zhu-Hong You, Xing Chen, De-Shuang Huang, Zheng-Wei Li, Gang Liu, Yin Wang

**Affiliations:** ^1^ School of Computer Science and Technology, China University of Mining and Technology, Xuzhou 21116, China; ^2^ Xinjiang Technical Institute of Physics and Chemistry, Chinese Academy of Science, Urumqi 830011, China; ^3^ School of Information and Electrical Engineering, China University of Mining and Technology, Xuzhou 221116, China; ^4^ School of Electronics and Information Engineering, Tongji University, Shanghai 201804, China; ^5^ College of Computer Science and Software Engineering, Shenzhen University, Shenzhen, Guangdong 518060, China

**Keywords:** disease, position-specific scoring matrix, protein self-interaction, cancer

## Abstract

Self-interacting Proteins (SIPs) play an essential role in a wide range of biological processes, such as gene expression regulation, signal transduction, enzyme activation and immune response. Because of the limitations for experimental self-interaction proteins identification, developing an effective computational method based on protein sequence to detect SIPs is much important. In the study, we proposed a novel computational approach called RVMBIGP that combines the Relevance Vector Machine (RVM) model and Bi-gram probability (BIGP) to predict SIPs based on protein sequence. The proposed prediction model includes as following steps: (1) an effective feature extraction method named BIGP is used to represent protein sequences on Position Specific Scoring Matrix (PSSM); (2) Principal Component Analysis (PCA) method is employed for integrating the useful information and reducing the influence of noise; (3) the robust classifier Relevance Vector Machine (RVM) is used to carry out classification. When performed on *yeast* and *human* datasets, the proposed RVMBIGP model can achieve very high accuracies of 95.48% and 98.80%, respectively. The experimental results show that our proposed method is very promising and may provide a cost-effective alternative for SIPs identification. In addition, to facilitate extensive studies for future proteomics research, the RVMBIGP server is freely available for academic use at http://219.219.62.123:8888/RVMBIGP.

## INTRODUCTION

Whether proteins can interact with their partners is a crucial problem for fundamental research. Self-interacting proteins (SIPs) is a special type of PPIs. SIPs are those whose more than two copies can interact with each other. Two SIP partners can be represented by the same gene and are the same copies, which can result in the formation of homo-oligomer. Knowledge of SIPs whether can play an important role in biological process and provides insight into the regulation of protein function and brings about a better understanding of disease mechanisms. During the past decade, it has been proved that homo-oligomerization play a key function in a wide range of biological processes by many researches [1], for instance, signal transduction [1], enzyme activation [1], gene expression regulation and immune response [1]. In previous study, it is found that SIPs can variously prolong the function diversity of proteins without increasing the size of genome. Thus, it is a powerful incentive for developing robust and effective computational methods for identifying SIPs based on protein sequence.

In recent years, a number of computational approaches proposed to predict PPIs. Such as, R Jansen *et al.* [2] proposed a method employing Bayesian networks for predicting protein-protein interactions genome-wide on yeast dataset, which obtained good prediction results. A Benhur *et al.* [3] proposed a kernel method to predict PPIs using protein sequences, which converts a kernel between single proteins into a kernel between pairs of proteins. The effectiveness of the method was evaluated using support vector machine classifier. Zahiri J *et al.* [4] proposed a computational method named as PPIevo to detect PPIs. The evolutionary information can be captured from PSSM (Position-Specific Scoring Matrix) of protein sequence employing the PPIevo approach. J Shen *et al.* [5] presented an approach to predict PPI by using only protein sequence's information. The approach employed a machine learning algorithm (support vector machine). These methods usually consider for the correlational information between protein pairs, for instance, co-expression, co-localization and coevolution [1]. However, this information is not available for detecting SIPs. In addition, the datasets that not contain SIPs used to predict PPIs. Because of these reasons, these computational methods are not fit for detecting SIPs. N Zaki *et al.* [6] proposed an approach called as PPI-PS (Pairwise Similarity) to predict PPIs. The PPI-PS combined pairwise similarity score with support vector machine (SVM) for detecting PPIs. The PPI-PS obtained reasonable experimental results for predicting PPIs. In the past study, Liu *et al.* [7] proposed a method integrating several representative known properties to create a prediction mode called as SLIPPER to predicting SIPs. There exists a variously disadvantage that the method can only dispose of these proteins that the current *human* interatomic contains. Due to the limitations of the aforementioned methods, there exists a critical challenge to develop automated methods for SIPs detection.

In the paper, we presented a novel computational approach called RVMBIGP to detect SIPs only using protein amino acids sequence. The proposed model generally can be divided into three steps: (1) an effective feature extraction method named BIGP is used to represent candidate self-interacting proteins by exploring evolutionary information embedded in PSI-BLAST-constructed PSSM; (2) PCA (Principal Component Analysis) is employed to decrease the dimensional of feature vectors and capture the useful information, which can decrease the effects of noise; (3) the robust classifier Relevance Vector Machine is employed to carry out classification. The fivefold cross validation is used in the experiment. These experimental results display that our RVMBIGP model can achieve very high accuracies of 95.48% and 98.80% on *yeast* and *human* datasets, respectively. In order to evaluate the performance of RVMBIGP, we also compared it with SVM classifier (support vector machine) and other several approaches on *yeast* and *human* datasets. It can be seen that proposed matrix-based feature representation can extract the hidden key information beyond the sequence itself and, hence, can yield much better prediction accuracy than previous method. It is demonstrated that our approach is fit for SIPs detection and can perform incredibly well for predicting SIPs.

## RESULTS AND DISCUSSION

### Performance of the proposed method

For demonstrating the effectiveness of our prediction model called as RVMBIGP, the experiment was executed on yeast and human dataset, respectively. To prevent the overfitting of the proposed approach, we divided yeast and human datasets into training datasets and independent test datasets respectively. More specifically, 1/6 of *human* dataset were randomly selected as independent test dataset and the remaining *human* dataset selected as training dataset. The same strategy was also used to apply in the *yeast* dataset. In addition, to provide a fair comparison, the experimental dataset was repeatedly constructed five times. In order to guarantee the fair, the parameters of RVMBIGP prediction model should be optimized. In the experiments, the Gaussian kernel function was selected and three parameters set up as following: beta = 0, initapla = 1/*N*, and width = 2, where width is Gaussian function's width, *N* represents a total of training dataset, and beta represents classification. The prediction model is report Ac, Sn, Pe and Mcc for *yeast* and *human* dataset. The results are displayed in Tables 1–2.

**Table 1:** Prediction performance of proposed method on yeast dataset by five tests

Testing set	Ac (%)	Sn (%)	Pe (%)	Mcc (%)
1	94.79	69.39	79.31	70.37
2	95.66	74.17	86.41	78.53
3	95.37	68.00	91.40	77.13
4	95.75	72.73	88.89	78.86
5	95.85	80.00	84.75	80.81
**Average**	**95.48 ± 0.42**	**72.86 ± 4.70**	**85.07 ± 6.73**	**77.14 ± 4.01**

**Table 2:** Prediction performance of proposed method on human dataset by five tests

Testing set	Ac (%)	Sn (%)	Pe (%)	Mcc (%)
1	98.90	89.86	95.12	91.94
2	98.93	92.77	93.97	92.86
3	98.83	91.80	94.12	92.40
4	98.45	87.92	94.72	90.54
5	98.90	89.87	96.38	92.54
**Average**	**98.80 ± 0.20**	**90.44 ± 1.89**	**94.86 ± 0.97**	**92.06 ± 0.91**

We can see from Table 1 that the average accuracies of five experiments are all above 94% for *yeast* dataset. Specifically, the each time overall accuracies of 94.79%, 95.66%, 95.37%, 95.75% and 95.85 were achieved. At the same time, the proposed method also obtained average Sensitivity, Precision, and Mcc of 72.86%, 85.07%, 77.14% and the standard deviations of them of 4.7%, 6.7%, and 4.0% on *yeast* dataset. Similarly average Accuracy of 98.80% was also obtained on *human* dataset. The average Mcc, Precision and Sensitivity of 92.06%, 94.86% and 90.44% and the standard deviations of them of 0.97%, 0.91% and 1.89% were also acquired respectively.

Because of the choice of feature extraction method and classifier, we can found from Table 1 and Table 2 that the proposed prediction model obtained very reasonable experimental results for predicting SIPs. The proposed feature extraction method play an important role for improving the prediction accuracy, which may be attributing to as following three reasons: (1) PSSM's advantage make it can capture useful information from protein sequence; (2) From biological perspective, the BIGP feature extraction method can describe the subsequence of protein sequence in the conserved areas. When this done, thus each protein sequence can obtain a set of bi-grams from the conserved area [8]. As a result, it can provide a greet help in predicting SIPs. (3) We converted into the dimensional of each BIGP feature vector from 400 to 350 through employing Principal Component Analysis (PCA) method for reducing the influence of noise. Thus, the experiment results show that the proposed approach may provide a useful tool for the accurate prediction of SIPs.

### Comparison with the SVM-based method

Despite our prediction model obtained god prediction results. However, for further evaluating the prediction performance of the proposed classifier, the comparison of prediction accuracy executed between RVM classifier and the SVM classifier (support vector machine) by using BIGP feature extraction approach on *human* and *yeast* dataset. The SVM classifier used the LIBSVM tool [9] to carry out classification. The RBF function (radial basis function) was choose as SVM's kernel function. A grid search method was employed to optimize the RBF kernel parameters, where c = 0.1 and g = 0.01.

**Figure 1:**
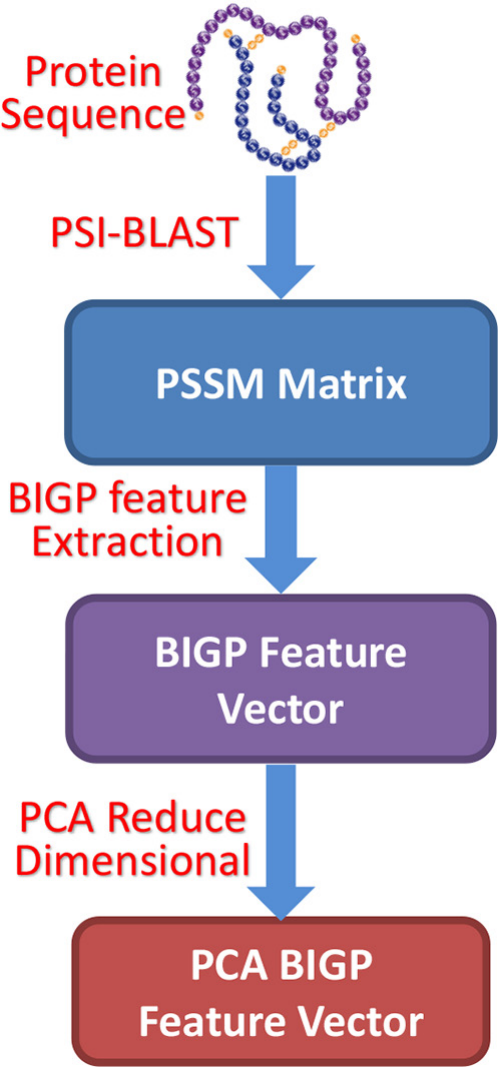
Flowchart of the proposed featureextraction method based on PSI-BLAST-constructed position specificscoring matrix

**Figure 2:**
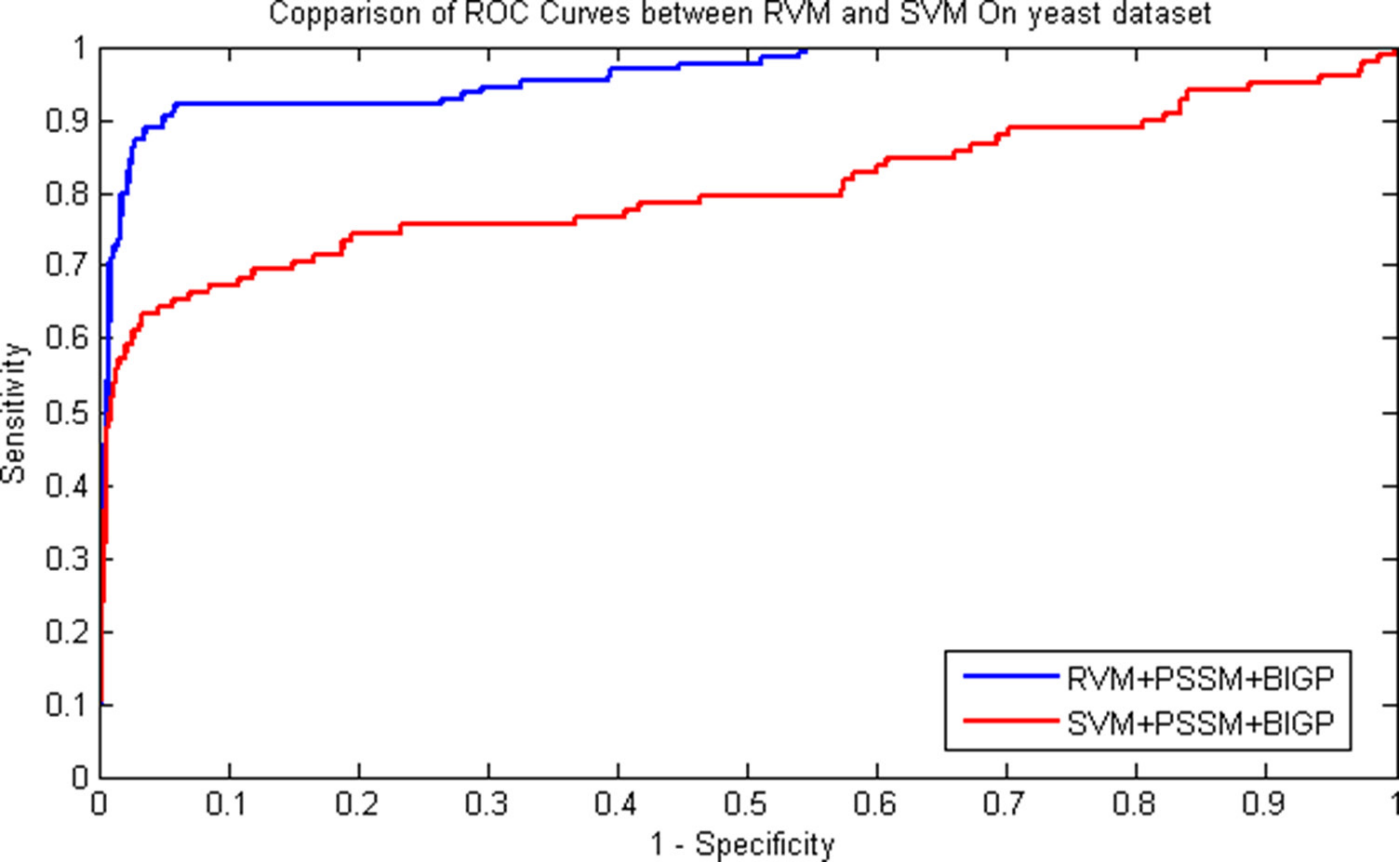
Performance comparisons betweenRVM and SVM on *yeast* dataset

The prediction results of SIPs for RVM and SVM classifier were presented in Table 3 and Table 4 on *yeast* and *human* datasets respectively. Similarity, the comparison of ROC Curves was shown in Figure 1 and Figure 2 on *yeast* and *human* datasets respectively. We can find from Table 3 that SVM obtained 91.35% average accuracy on *yeast* dataset. However, the RVM classifier achieved 95.48% average Accuracy. Similarly as displayed in Table 4, 98.80% average Accuracy obtained by the proposed RVM classifier and 95.35% average Accuracy achieved by the SVM classifier on *human* dataset. These prediction results from Table 3 and Table 4 demonstrated that the performance of RVM is obviously higher than that of SVM. Meanwhile, it can be found from Figure 1 and Figure 2, RVM's ROC curves is also obviously better than that of SVM. This may be attributed to as following reason: (1) The RVM classifier can greatly reduce kernel function calculation; (2) The obvious disadvantage of SVM that kernel function need to be meet the demand of Mercer overcome by RVM classifier. As a result, all of these demonstrated that the proposed prediction model might become useful tools for predicting SIPs, as well as other bioinformatics tasks.

**Table 3:** Comparison of the prediction performance by the RVM and SVM classifier based on BIGP on the yeast dataset

Testing set	Ac (%)	Sn (%)	Pe (%)	Mcc (%)
RVM+PSSM+BIGP				
1	94.79	69.39	79.31	70.37
2	95.66	74.17	86.41	78.53
3	95.37	68.00	91.40	77.13
4	95.75	72.73	88.89	78.86
5	95.85	80.00	84.75	80.81
Average	95.48 ± 0.42	72.86 ± 4.70	85.07 ± 6.73	77.14 ± 4.01
SVM+PSSM+BIGP				
1	92.86	29.59	85.29	49.87
2	90.93	22.50	96.43	44.52
3	89.77	20.00	80.65	38.99
4	91.31	25.62	100.0	48.30
5	91.89	35.20	93.62	55.25
Average	91.35 ± 1.14	26.58 ± 6.00	91.20 ± 8.01	47.21 ± 5.99

**Table 4:** Comparison of the prediction performance by the RVM and SVM classifier based on BIGP on the human dataset

Testing set	Ac (%)	Sn (%)	Pe (%)	Mcc (%)
RVM+PSSM+BIGP				
1	98.90	89.86	95.12	91.94
2	98.93	92.77	93.97	92.86
3	98.83	92.80	94.12	92.40
4	98.45	87.92	94.72	90.54
5	98.90	89.87	96.38	92.54
Average	98.80 ± 0.20	90.44 ± 1.89	94.86 ± 0.97	92.06 ± 0.91
SVM+PSSM+BIGP				
1	95.44	39.63	98.85	61.14
2	95.58	46.81	97.35	66.02
3	95.41	48.36	94.40	66.11
4	94.85	44.53	98.33	64.43
5	95.51	50.21	90.84	66.23
Average	95.35 ± 0.30	45.91 ± 4.08	95.95 ± 3.33	64.79 ± 2.17

### Comparison with other methods

In the paper, for further evaluating the performance of the proposed prediction model, the comparison of prediction performance executed between the proposed prediction model called RVMBIGP and other existing methods: SPAR, CRS, SLIPPER, DXECPPI [10], PPIevo [4] and LocFuse [11] based on the *yeast* and *human* dataset. These results were displayed in Table 5 and Table 6 using the above mentioned methods on *yeast* and *human* datasets. From Table 5, we can found that the proposed model average accuracy is obviously better other methods on *yeast* dataset. Similarity, as shown in Table 6, the prediction results of our final model is also obviously better other methods on *human* dataset. The results demonstrated that the RVMBIGP prediction model has good executive ability. This further proved that our prediction model is a useful tool for SIPs prediction.

**Table 5:** Performance comparison of the RVMBIGP and the other methods on yeast dataset

Model	Ac (%)	Sp (%)	Sn (%)	Mcc (%)
SLIPPER [7]	71.90	72.18	69.72	28.42
DXECPPI [10]	87.46	94.93	29.44	28.25
PPIevo [4]	66.28	87.46	60.14	18.01
LocFuse [11]	66.66	68.10	55.49	15.77
CRS [1]	72.69	74.37	59.58	23.68
SPAR [1]	76.96	80.02	53.24	24.84
**Proposed method**	**95.48**	**98.37**	**72.86**	**77.14**

**Table 6:** Performance comparison of the RVMBIGP and the other methods on human dataset

Model	Ac (%)	Sp (%)	Sn (%)	Mcc (%)
SLIPPER [7]	91.10	95.06	47.26	41.97
DXECPPI [10]	30.90	25.83	87.08	8.25
PPIevo [4]	78.04	25.82	87.83	20.82
LocFuse [11]	80.66	80.50	50.83	20.26
CRS [1]	91.54	96.72	34.17	36.33
SPAR [1]	92.09	97.40	33.33	38.36
**Proposed method**	**98.80**	**99.56**	**90.44**	**92.06**

## MATERIALS AND METHODS

### Dataset

There are 20,199 curated *human* protein sequences in the UniProt database [12]. We can obtain the PPI datasets from variously resources, containing DIP [13], BioGRID [14], IntAct [15], InnateDB [16] and MatrixDB [17]. In the work, the PPIs datasets were created, which only contain the identical two interactions protein sequences. The interaction type of PPIs datasets was defined as ‘direct interaction’ in relevant databases. As a result, 2994 human Self-interaction protein sequences obtained in the experiment. For assessing the efficiency of our prediction model, we created the experiment datasets through the following three steps [1]: (1) We only reserved the protein sequences, whose length longer than 50 residues and less than 5000 residues from the whole *human* proteome; (2) The Protein Self-interaction data were selected for constructing positive datasets, which must be meet one of the following conditions: (a) The Self-interaction positive protein datasets have been found through at least two kinds of large scale experiments or one small-scale experiment; (b) the protein has been defined as homooligomer (including homodimer and homotrimer) in UniProt; (c) The Self-interaction positive protein datasets have been reported by at least two publications; (3) For creating the negative dataset, we removed all types of SIPs from the whole *human* proteome (including proteins annotated as ‘direct interaction’ and more extensive ‘physical association’) and UniProt database. Thus, 1441 human positive SIPs and 15,938 *human* negative non-SIPs were created in the experiment. In addition, for further proving the prediction performance of RVMBIGP, the yeast dataset that contains 710 positive SIPs and 5511 negative non-SIPs was constructed by using the same strategy [1].

### Position specific scoring matrix

Position Specific Scoring Matrix (PSSM) was originally used to detect distantly related proteins. Now, PSSM is employed to predict protein disulfide connectivity, quaternary structural attributes, and folding pattern [18]. In the paper, we used PPSM to predict SIPs. Using the Position Specific Iterated BLAST (PSI-BLAST) [19] transform each protein sequence into a PSSM matrix. A PSSM is an *N* × 20 matrix, ***M* = {*M _ij_ i* : 1 = 1…*N*, *j* = 1…20}**, where *N* is the length of a given protein sequence, and 20 are a total of 20 amino acids and can assign the score *M_ij_* that represent the *j_th_* amino acid in the *i_th_* position for the query protein sequence. The score *M_ij_* is, $\;M_{ij}=\overset{20}{\underset{k=1}{\sum }}\text{​ }p(i,k)\times q(j,k)$ where *p(i, k)* represents the appearing frequency value of the *k_th_* amino acid at position *i* of the probe, and *q(i, k)* is the value of Dayhoff's mutation matrix between *j_th_* and *k_th_* amino acids. Thus, a high score represents a well conserved position and a low score represents a weakly conserved position.

In our work, in order to create experiment datasets, we used PSI-BLAST to convert each protein sequence into a PSSM for predicting SIPs. For obtaining highly and widely homologous sequences, we set up the e-value parameter of PSI_BLAST is 0.001 and selected three iterations. Finally, the PSSM can be expressed as a 20-dimensional matrix though using PSI-BLAST, which contains *M*× 20 elements, where *M* is the number of residues of a protein and 20 columns represent a count of 20 amino acids.

### Bi-gram probabilities

The Bi-gram Probabilities (BIGP) have been used for protein fold recognition. In the literature [20], a given protein sequence was represented using its original primary sequence or its consensus sequence. Instead of, we employed the improved BIGP feature extraction method that proposed by the literature [21] and expressed a protein sequence by its PSSM (PSSM has been mentioned in the 2.2 section of the paper) directly for predicting SIPs. In detail, the Bi-gram feature vector was computed through counting the bi-gram frequencies of occurrences in PSSM. It is assumed that P represents the PSSM of a protein sequence, which contains *L* rows and 20 columns, where *L* represents the length of a given protein sequence length and 20 columns represents a total of 20 amino acids. The PSSM element *P_ij_* can be interpreted as the relative probability of *j_th_* amino acid at the *i_th_* location of the primary protein sequence, *P_ij_* can be expressed as $\;P_{ij}=\overset{20}{\underset{j=1}{\sum }}i:1=1\ldots L,j=1\ldots 20$. The frequency of occurrence of transition from m_th_ amino acid to n_th_ amino acid can be defined as following:

(1) $${\text{BIGP}}_{\text{mn}}=\overset{L-1}{\underset{i=1}{\sum }}P_{i,m}P_{i+1,n}1\leq m\leq 20,1\leq n\leq 20$$

The equation (1) gives 400 frequencies of occurrences BIGP_mn_ for 400 bi-gram transitions, the matrix BIGP called the bi-gram occurrence matrix, whose 400 elements represent the bi-gram feature vector [21] as following:

(2) $$\begin{array}{l} BF=[BGP_{1,1},BGP_{1,2}\ldots BGP_{1,20},BGP_{2,1},\\ \;\ldots BGP_{2,20},\ldots \ldots BGP_{20,1},\ldots BGP_{20,20}] \end{array}$$

These bi-gram features can also be expressed as following:

(3) $$BF=[\varphi_{1,},\varphi_{2},\varphi_{3,}\ldots \varphi_{u,},\ldots \varphi_{\theta }]$$

Where *θ* = mn = 400 is the dimensionality of the feature vector **BF**, the φ_u_ can be represented as following:

(4) $$\varphi_{u}=\{\begin{array}{c} BGP_{1,u}\;\;\;\;\;\;\;\;\;\;\;\;\;\;\;\;\;\;\;\;\;\;\;\;\;\;\;\;\;(1\leq u\leq 20)\\ BGP_{2,u-20\;}\;\;\;\;\;\;\;\;\;\;\;\;\;\;\;\;\;\;\;\;(21\leq u\leq 40)\\ \ldots \ldots \;\;\;\;\;\;\;\;\;\;\;\;\;\;\;\;\;\;\;\;\;\;\;\;\;\;\;\;\;\;\;\;\;\;\;\;\;\;\;\;\;\;\;\;\;\;\;\;\\ BGP_{20,u-380\;}\;\;\;\;\;\;\;\;\;\;\;\;(381\leq u\leq 400) \end{array}$$

**Figure 3:**
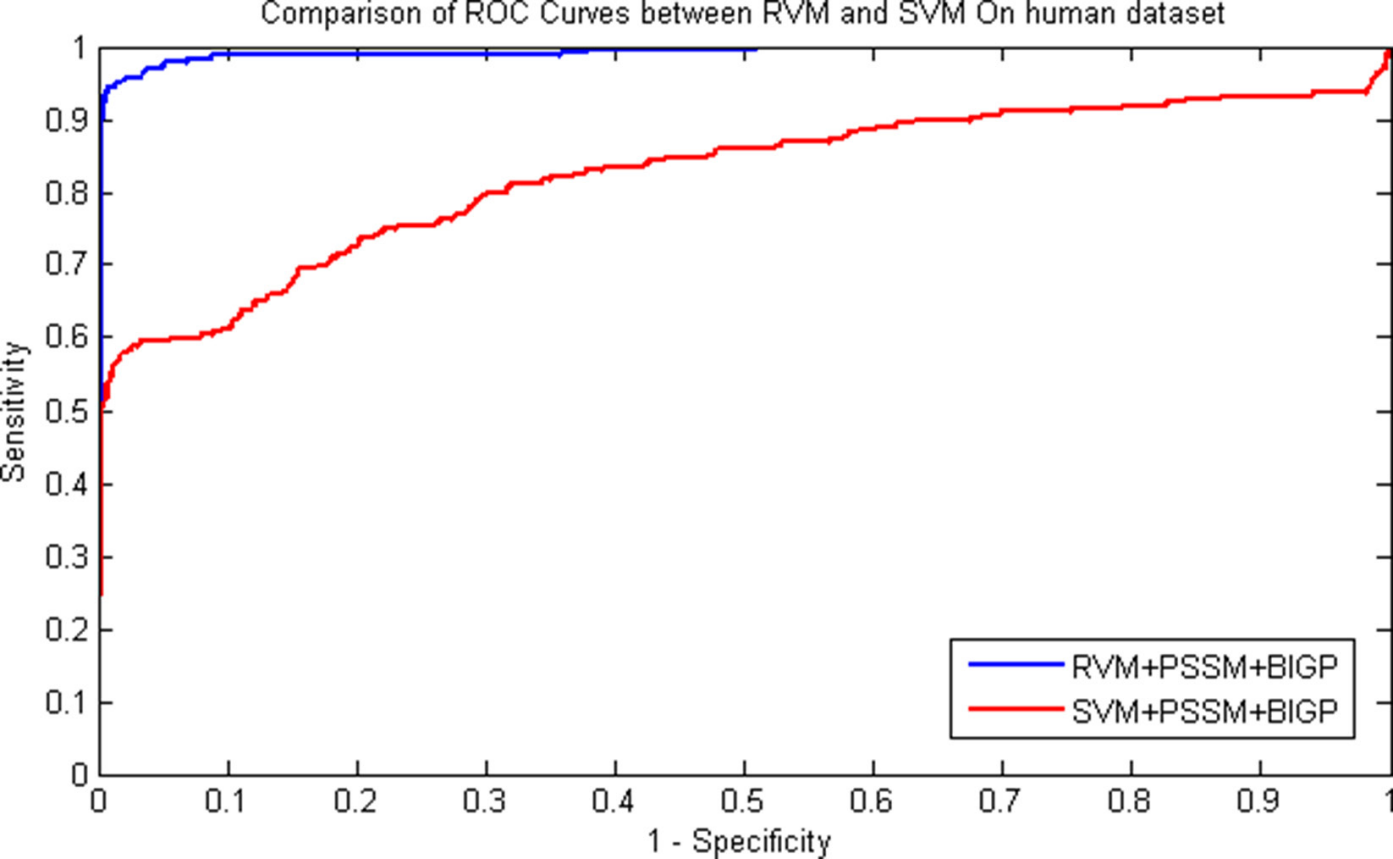
Performance comparisons betweenRVM and SVM on *human* dataset

Finally, each *yeast* and *human* protein sequence was transformed into a 400-dimensional vector using the Bi-gram Probabilities feature extraction method. In our work, in order to reduce the influence of noise and improve the prediction accuracy, the dimensional of *yeast* and *human* were reduced from 400 to 350 by using Principal Component Analysis (PCA) method. The flow chart of the proposed feature extraction method is displayed in Figure 3.

### Relevance vector machine

The characteristics of the Relevance Vector Machine described in the literature [22]. We assumed ${\left\{x_{n},t_{n}\right\}}_{n=1}^{N}$, $x_{n}\in R^{d}$ represents the training set for binary classification problems, where $t_{n}\in \{0,1\}$ is the training set label, *t_i_* is the testing set label, and $t_{i}=y_{i}+\varepsilon_{i}$ where $y_{i}=w^{T}\varphi \left(x_{i}\right)=\sum_{j=1}^{N}w_{j}K\left(x_{i},x_{j}\right)+w_{0}$ is the classification model; *ε_i_* is the additional noise, with a mean value of zero and a variance of *σ^2^*, where $\varepsilon_{i}\sim N\left(0,\sigma^{2}\right),t_{i}\sim N\left(y_{i},\sigma^{2}\right)$. It is assumed that the training sets are independent and identically distributed; the vector *t* submits to as following distribution:

(2) $$p\left(t|x,w,\sigma^{2}\right)={\left(2\pi \sigma^{2}\right)}^{-N/2}\text{exp}[-\frac{1}{2\sigma^{2}}|\left|t-\varphi w\right|{|}^{2}]$$

Where *φ* is defined as following:

(3) $$\varphi =\left(\begin{array}{ccc} 1 & k(x_{1},x_{1})\cdots & k(x_{1},x_{N}))\\ \ldots & \ldots & \ldots \\ 1 & k\left(x_{N},x_{1}\right)\ldots & k(x_{N},x_{N}) \end{array}\right)$$

The training set label *t* is employed to detect the testing set label t_*_, given by

(4) $$p\left(t_{*}|t\right)=\int^{\text{​}}p\left(t_{*}|w,\sigma^{2}\right)p(w,\sigma^{2}|t)dwd\sigma^{2}$$

Because of making the value of most components of the weight vector *w* zero and reducing the number of calculation of the kernel function, additional conditions is attached to the weight vector *w*. Assuming that *w_i_* obeys a distribution with a mean value of zero and a variance of $\alpha_{i}^{-1}$, the mean $\;w_{i}\sim N(0,\;\alpha_{i}^{-1})$, $p\left(w|a\right)=\prod_{i=0}^{N}p(w_{i}|a_{i})$, where *a* is a hyper-parameters vector of the prior distribution of the weight vector *w*.

(5) $$\begin{array}{l} p\left(t_{*}|t\right)=\int^{\text{​}}p\left(t_{*}|w,a,\sigma^{2}\right)p(w,a,\sigma^{2}|t)\\ dwdad\sigma^{2} \end{array}$$

(6) $$p\left(t_{*}|w,a,\sigma^{2}\right)=N(t_{*}\;|y\left(x_{*};w\right),\sigma^{2})$$

Because $p\left(w,a,\sigma^{2}|t\right)$ cannot be obtained by an integral. As a result, it must be resolved using a Bayesian formula, as given

(7) $$p\left(w,a,\sigma^{2}|t\right)=p\left(w|a,\sigma^{2},t\right)p(a,\sigma^{2}|t)$$

(8) $$p\left(w|a,\sigma^{2},t\right)=p\left(t|w,\sigma^{2}\right)p\left(w|a\right)/\text{​}p\left(t|a,\sigma^{2}\right)$$

The integral of the product of $p(t|a,\sigma^{2})$ and p(w|a) as following:

(9) $$p\left(t|a,\sigma^{2}\right)={\left(2\pi \right)}^{-N/2}|\Omega {|}^{-1/2}\text{exp}(-\frac{t^{T}\Omega^{-1}t}{2})$$

(10) $$\Omega =\sigma^{2}I+\varphi A^{-1}\varphi^{T},\;A=diag\left(a_{0,}a_{1,}\ldots ,a_{N}\right)$$

(11) $$p\left(w|a,\sigma^{2},t\right)={\left(2\pi \right)}^{-(N+1)/2}|\Sigma {|}^{-1/2}\text{exp}(-\frac{{\left(w-u\right)}^{T}\left(w-u\right)}{2})$$

(12) $$\Sigma ={\left(\sigma^{-2}\varphi^{T}\varphi +A\right)}^{-1}$$

(13) $$u=\sigma^{-2}\Sigma \varphi^{T}t$$

For the sake of $p(a,\sigma^{2}|t)\infty \;p(t|a,\sigma^{2})$ $p(a)p(\sigma^{2})$ and $\;p(a,\sigma^{2}|t$ cannot be solved by means of integration, the solution is approximated using the maximum likelihood method, represented by

(14) $$\left(a_{MP},\sigma_{MP}^{2}\right)=\underset{\;\;\;a,\sigma^{2}}{\arg}maxp(t|a,\sigma^{2})$$

The iterative process of *a_mp_* and *σ*^2^*_mp_* given by:

(15) $$\{\begin{array}{c} a_{i}^{new}=\frac{\gamma_{i}}{\mu_{i}^{2}}\\ {\left(\sigma^{2}\right)}^{new}=\frac{|\left|t-\varphi \mu \right|{|}^{2}}{N-\sum_{i=0}^{N}\mu_{i}}\\ \gamma_{i}=1-a_{i}\sum^{\text{​}}i,i \end{array}$$

Here $\sum^{\text{​}}i,i$ is *i*th element in the Σ's diagonal and the initial value of *a* and *σ*^2^ can be decided via the approximation of *a_mp_* and $\sigma_{MP}^{2}$ using formula (15) continuously updated. After enough iterations, most of a*_i_* will be close to infinity, the corresponding parameters in *w_i_* will be zero, and other a*_i_* values will be close to finite. The resulting corresponding parameters *x_i_* of a*_i_* are now referred to as the relevance vector.

### Performance evaluation

In the paper, in order to evaluate power of the proposed method, the following measures are used to assess the performance of the RVM classifiers employed in this work. The definition is showed as following:

$$\begin{array}{ccc} \text{Ac} & = & \frac{\text{TP}+\text{TN}}{\text{TP}+\text{FP}+\text{TN}+\text{FN}}\\ \text{Sn} & = & \frac{\text{TP}}{\text{TP}+\text{TN}}\\ \text{Sp} & = & \frac{\text{TN}}{\text{FP}+\text{TN}}\\ \text{Pe} & = & \frac{\text{TP}}{\text{FP}+\text{TP}} \end{array}$$

$$\mathrm{Mcc}=\frac{\left(\text{TP}\times \text{TN})-(\text{FP}\times \text{FN}\right)}{\sqrt{\left(\text{TP}+\text{FN})\times (\text{TN}+\text{FP})\times (\text{TP}+\text{FP})\times (\text{TN}+\text{FN}\right)}}$$

Where Ac represents Accuracy, Sn represents Sensitivity, Sp is specificity, Pe is Precision and Mcc represents Matthews's correlation coefficient respectively. In the above formula, TP represents true positives (the count of true interacting pairs correctly predicted), FP represents false positives (the count of true non-interacting pairs falsely predicted), TN represents true negatives (the number of true non-interacting pairs predicted correctly) and FN represents false negatives (true interacting pairs falsely predicted to be non-interacting pairs). Moreover, in order to assess the performance of the proposed prediction model, we created Receiver Operating Curve (ROC) in the experiment.

### Webserver

In order to provide convenience for using the proposed prediction model, a web server created which executes the prediction function of the proposed RVMBIGP model. It is available at http://219.219.62.123:8888/RVMBIGP/. The Web Server mainly used to predict SIPs on *human* dataset, which enable users can obtain the probability scores of SIPs by RVMBIGP prediction model. The prediction results can be listed on the Webpage and send it to the users’ email.

## CONCLUSIONS

In the paper, we proposed an approach named RVMBIGP was proposed, which combines the RVM (Relevance Vector Machine) model with BIGP (Bi-gram probability) to predict SIPs based on protein sequence information. There are several obvious advantages for the proposed method: (1) an effective feature extraction method named BIGP is used to represent protein sequences on PSSM, which can characterize the subsequence of amino acids in the conserved regions and capture the useful evolutionary information; (2) PCA (Principal Component Analysis) method employed to capture the evolutionary information and reduce the influence of noise; (3) using the robust classifier Relevance Vector Machine (RVM) to carry out classification. When performed on *yeast* and *human* datasets, the proposed RVMBIGP model obtained high accuracy of 95.48% and 98.80%, respectively, which obviously higher than the prediction model based SVM classifier and other exiting methods. In conclusion, the proposed RVMBIGP prediction model is robust, powerful and effective. This make it is a useful tool and suitable for predicting SIPs, as well as other bioinformatics tasks. More machine learning algorithms and effective feature extraction approaches should be developed for identifying SIPs in the future study.
